# Assessment of a Personal Interactive Carbon Monoxide Breath Sensor in People Who Smoke Cigarettes: Single-Arm Cohort Study

**DOI:** 10.2196/22811

**Published:** 2020-10-02

**Authors:** Jennifer D Marler, Craig A Fujii, Kristine S Wong, Joseph A Galanko, Daniel J Balbierz, David S Utley

**Affiliations:** 1 Carrot Inc. Redwood City, CA United States; 2 Biostatistics Core for the Center for Gastrointestinal Biology and Disease and the biostatistician for the Clinical Nutrition Research Center Department of Medicine, Division of Gastroenterology and Hepatology University of North Carolina at Chapel Hill Chapel Hill, NC United States

**Keywords:** smoking cessation, digital health, smartphone, digital sensor, carbon monoxide, breath sensor, biofeedback

## Abstract

**Background:**

Tobacco use is the leading cause of preventable morbidity and mortality. Existing evidence-based treatments are underutilized and have seen little recent innovation. The success of personal biofeedback interventions in other disease states portends a similar opportunity in smoking cessation. The Pivot Breath Sensor is a personal interactive FDA-cleared (over-the-counter) device that measures carbon monoxide (CO) in exhaled breath, enabling users to link their smoking behavior and CO values, and track their progress in reducing or quitting smoking.

**Objective:**

The objective of this study is to assess the Pivot Breath Sensor in people who smoke cigarettes, evaluating changes in attitudes toward quitting smoking, changes in smoking behavior, and use experience.

**Methods:**

US adults (18-80 years of age, ≥10 cigarettes per day [CPD]) were recruited online for this remote 12-week study. Participants completed a screening call, informed consent, and baseline questionnaire, and then were mailed their sensor. Participants were asked to submit 4 or more breath samples per day and complete questionnaires at 1-4, 8, and 12 weeks. Outcomes included attitudes toward quitting smoking (Stage of Change, success to quit, and perceived difficulty of quitting), smoking behavior (quit attempts, CPD reduction, and 7-, 30-day point prevalence abstinence [PPA]), and use experience (impact and learning).

**Results:**

Participants comprised 234 smokers, mean age 39.9 (SD 11.3) years, 52.6% (123/234) female, mean CPD 20.3 (SD 8.0). The 4- and 12-week questionnaires were completed by 92.3% (216/234) and 91.9% (215/234) of participants, respectively. Concerning attitude outcomes, at baseline, 15.4% (36/234) were seriously thinking of quitting in the next 30 days, increasing to 38.9% (84/216) at 4 weeks and 47.9% (103/215) at 12 weeks (both *P*<.001). At 12 weeks, motivation to quit was increased in 39.1% (84/215), unchanged in 54.9% (118/215), and decreased in 6.0% (13/215; *P*<.001). Additional attitudes toward quitting improved from baseline to 12 weeks: success to quit 3.3 versus 5.0 (*P*<.001) and difficulty of quitting 2.8 versus 4.3 (*P*<.001). Regarding smoking behavior, at 4 weeks, 28.2% (66/234) had made 1 or more quit attempts (≥1 day of abstinence), increasing to 48.3% (113/234) at 12 weeks. At 4 weeks, 23.1% (54/234) had reduced CPD by 50% or more, increasing to 38.5% (90/234) at 12 weeks. At 12 weeks, CPD decreased by 41.1% from baseline (*P*<.001), and 7- and 30-day PPA were 12.0% (28/234) and 6.0% (14/234), respectively. Concerning use experience, 75.3% (171/227) reported the sensor increased their motivation to quit. More than 90% (>196/214) indicated the sensor taught them about their CO levels and smoking behavior, and 73.1% (166/227) reported that seeing their CO values made them want to quit smoking.

**Conclusions:**

Use of the Pivot Breath Sensor resulted in a significant increase in motivation to quit, a reduction in CPD, and favorable quit attempt rates. These outcomes confer increased likelihood of quitting smoking. Accordingly, the results support a role for biofeedback via personal CO breath sampling in smoking cessation.

**Trial Registration:**

ClinicalTrials.gov NCT04133064; https://clinicaltrials.gov/ct2/show/NCT04133064

## Introduction

Cigarette smoking is the largest preventable cause of morbidity and mortality, accounting for approximately 480,000 annual deaths in the United States, including about 30% of cancer deaths and 30% of cardiovascular disease deaths [1-3]. Smoking remains a pervasive public health problem with a prevalence of 13.7% (34.2 million people) in 2018 [4]. Efforts to advance smoking cessation are a top priority. Launched by the US government in 2010, the 2020 Healthy People initiative identified several related goals including decreasing the prevalence of smoking to 12% or less (tobacco use objective 1.1, or TU-1.1), increasing the proportion of US adults who attempt to quit smoking cigarettes to 80.0% or more (TU-4.1), and increasing recent smoking cessation success to 8.0% or more (TU-5.1) [5]. As a check-in, half way through the 2020 Healthy People agenda in 2015, 68.0% of adult smokers wanted to stop smoking, 55.4% made a past-year quit attempt, and 7.4% recently quit smoking [6,7].

Evidence-based interventions proven to increase quit rates include counseling (individual, group, or phone) and FDA-approved pharmacotherapy. While efficacious, the success of these interventions has been limited by challenges with access, desirability, and convenience, with less than a third of individuals using counseling or medication during quit attempts [6].

Accordingly, smoking cessation is ripe for new technology and approaches. The management of other disease states such as overweight/obesity, hypertension, and diabetes have included the application of personal devices that provide biofeedback, such as wearable activity trackers (eg, Fitbit, Garmin, Jawbone), home-use blood pressure monitors, and continuous glucose monitoring and insulin pumps. These technologies are associated with improved outcomes [8-13], and have a common thread of enabling the user to quantify and monitor personal disease-specific metrics and track progress toward associated goals.

The successes of these novel approaches raise the question of a possible role of personal biofeedback in smoking cessation. One such type of biofeedback is carbon monoxide (CO), a product of the combustion process of smoking. During smoking, CO enters the lungs and crosses pulmonary capillaries to enter the blood stream, where it binds to heme in red blood cells. CO is eliminated from the body by exhalation. Exhaled CO, measured in parts per million (ppm), can be quantified and tracked using a CO breath sensor. With a half-life of approximately 4-5 hours, exhaled CO is well-suited for tracking changes over relatively short periods; once an individual stops smoking, exhaled CO decreases, returning to nonsmoking levels within approximately 24 hours [14].

In “The Tobacco Dependence Treatment Handbook: A Guide to Best Practices,” Abrams et al [15] report that, “providing individualized feedback about changes in personal levels of carbon monoxide before and after smoking is a powerful message that encourages individuals to make a quit attempt.” Further, Abrams notes that, “In the context of smoking cessation treatment, carbon monoxide levels” and other biomarker feedback “can be utilized to demonstrate the impact of smoking on the smoker and his/her family.”

Beard et al [16] conducted work in this area by providing a personal, mobile CO breath sensor to smokers and asking them to use the monitor regularly throughout the day for 6 weeks, with the goal of maintaining their CO level below 10 ppm. During the first 2 weeks, participants were instructed to record daily their cigarette consumption, usage of the CO monitor and any nicotine replacement therapy, average CO levels, and whether they had attempted to keep their reading below 10 ppm. The participants were not told to quit and were not specifically seeking a quit program. Participants (n=10, 5 males, average age 48.6 years) used the monitor an average of 3 times per day. Average daily cigarette consumption decreased from 14.1 (SD 6.03) at baseline to 9.8 (SD 4.95; *P*=.036) during the 2 weeks of daily CO monitoring and to 9.5 (SD 5.50; *P*=.127) at 6-week follow-up. At follow-up, 50% (5/10) of participants had attempted to quit smoking and one of these participants successfully quit. The majority of smokers reported that they found the CO monitor helpful (79.3%, n=111/140 responses) and that they felt as though the monitors had reduced their cigarette consumption (70%, 7/10 participants). The study investigators concluded that the use of the CO monitors was found to be acceptable and to increase motivation to consider a quit attempt.

In 2018, Patrick et al [17] reported results of a 9-day study of 41 participants using an FDA 510k-cleared mobile CO breath sensor, the Carbon Monoxide Breath Sensor System (COBSS) [17]. This study evaluated the first phase of the multiphase Pivot Smoking Cessation Program, designed to deliver the US Clinical Practice Guidelines for Treating Tobacco Use and Dependence. The focus of the evaluated program phase was to encourage the participants to explore their smoking behavior. Participants completed activities and had the opportunity to log cigarettes within the Pivot app, and interact with a coach via SMS text message–based interactions. More than 80% of participant (34-39 of 41) completed 1 or more CO breath samples each day, and more than 56% (23-27 of 41) completed 5 or more samples each day. In matched pair analyses, significant positive changes in mean attitudes toward quitting (scale 1-10) were evident from baseline (T1) to study exit (T2), including increased readiness to quit (T1 mean 6.1, T2 mean 7.4, *P*=.005), lower perceived difficulty (T1 mean 3.7, T2 mean 5.6, *P*=.001), and greater expectations of success (T1 mean 4.5, T2 mean 6.5, *P*<.001). At exit, 78% (32/41) of participants reported decreasing the number of cigarettes smoked per day during the study.

Marler et al [18] followed the aforementioned study with results in 319 smokers who underwent the complete Pivot program, which included a personal CO breath sensor, smartphone app, and in-app SMS text messaging–based human coaching. There were significant positive changes in attitudes during the prequit portion of the program, including increased confidence to quit (*P*<.001) and decreased expected difficulty maintaining quit (*P*<.001). Among the participants who completed the final questionnaire and reported the program increased their motivation to stop smoking (85.7%, 233/272), using the breath sensor was the most common reason for the increased motivation. At the end of the program, 7- and 30-day point prevalence abstinence (PPA) rates were 32.0% (102/319, intention to treat [ITT]) and 27.6% (88/319, ITT), respectively. Of those who did not achieve PPA, 25.9% (44/170) had reduced their cigarettes per day (CPD) by 50% or more.

Additional studies have explored the use of exhaled CO as a tool to add to quit programs to bolster motivation and support quit attempts and cessation. Results from these studies are mixed. Foulds et al [19] assessed outcomes 28 days after target quit date in 225 smokers randomized to receive motivational “Lung Age” feedback (exhaled CO values and forced expiratory volume over 1 second) versus minimal feedback. All participants were offered 6 weekly group coaching sessions and nicotine patches. Lung Age feedback did not improve quit rates or compliance with the program. Hajek et al [20] assessed outcomes in pregnant smokers who were randomized to receive midwife-delivered smoking cessation intervention (brief counseling, written materials, arrangements for self-help support, and feedback on exhaled CO levels) versus usual care. A significant difference was reported in those who had quit smoking in the 3 months prior to study start; they had a higher postdelivery PPA rate compared with those who were not recent quitters at the start of the study (65% vs 53%, *P*<.05). However, significant differences were not reported in other outcomes, such as continuous abstinence for at least 3 months prior to delivery or continuous abstinence from 3 months predelivery to 6 months postdelivery. The authors concluded the midwife-delivered intervention did not seem to be an effective method of helping pregnant smokers stop smoking.

Some studies showed favorable early outcomes that did not translate to longer-term results. In 160 smokers randomized to receive cessation leaflets and quit advice (usual care) versus usual care plus exhaled CO level feedback (intervention), Shahab et al [21] reported favorable short-term effects on the cognitive antecedents of smoking behavior and cessation in those who received the intervention. While the investigators reported a greater likelihood of cessation in the intervention group among those with higher self-efficacy, there were no differences in quit attempts or abstinence between the 2 groups at 6 months. McClure et al [22] assessed 536 smokers randomized to receive personally tailored feedback based on lung function, CO exposure, and smoking-related symptoms (experimental group) versus generic information about the risks of smoking and personalized counseling focused on diet, BMI, and physical activity (control group). All participants were advised to quit smoking and offered access to a free telephone smoking cessation counseling program. Immediately post-treatment, the experimental group rated themselves as more likely to try to quit (*P*=.02) and reported a greater mean increase in their motivation to quit than controls (*P*=.04). These group differences in motivation did not persist at 1-month follow-up. At 6- and 12-month follow-up, there was no greater motivation to quit, use of treatment services, or abstinence in the experimental group compared with controls [23]. Indeed, the control group had greater motivation to quit at 12 months, use of pharmacotherapy at 6 months, and 30-day PPA at 6 months.

And some studies report favorable longer-term results as well. Choi et al [24] randomized 95 adult male smokers to receive 5-10 minutes of smoking cessation education, undergo exhaled CO measurement, and complete questionnaires (intervention) versus receive self-help materials (control). At 4 weeks, motivation to quit was significantly improved in the intervention group (*P*=.03). In another randomized control trial including 98 smokers, home health nurses provided motivation enhancement (motivational interviewing and exhaled CO feedback) or standard care (AHCPR [Agency for Health Care Policy and Research] guidelines for smoking cessation) [25]. Individuals in the motivation enhancement group had more quit attempts and a greater reduction in CPD at follow ups through 12 months.

With the exception of Beard et al [16], the aforementioned studies incorporated exhaled CO as part of multicomponent smoking cessation programs. As a result, it is difficult to specifically identify the role of exhaled CO in reported motivation and smoking behavior outcomes. Moreover, with few exceptions [16-18], these exhaled CO measurements were obtained through health care providers or study personnel during study visits, with very few CO breath samples collected during these studies. It is, however, unclear how outcomes might differ when exhaled CO is regularly measured and tracked by smokers as personal biofeedback. Accordingly, this study sought to more directly focus on the potential role of exhaled CO when breath was sampled by smokers themselves using a personal mobile breath sensor, assessing changes in attitudes toward quitting smoking, changes in smoking behavior, and use experience.

## Methods

### Study Design

This was a prospective, open-label single-arm cohort study conducted with Institutional Review Board approval. The study was performed remotely on an ambulatory basis. Study participants were asked to set up the Pivot Breath Sensor and participate for 12 weeks with an emphasis on providing daily breath samples and completing online study questionnaires periodically throughout the study.

### Consent and Ethical Approval

All participants provided electronic informed consent before participation. The study was reviewed and approved by Solutions IRB (protocol number 2019/09/3), and registered with ClinicalTrials.gov (NCT04133064).

### Study Device

The Pivot Breath Sensor is a component of Pivot’s comprehensive evidence-based digital tobacco cessation solution, which also includes an interactive mobile Pivot app, lessons based in cognitive behavioral therapy and self-determination theory, nicotine replacement therapy, dedicated human coaching by tobacco treatment specialists via SMS text messaging, and a moderated online community. In keeping with this study’s focus on the impact and use experience of the Pivot Breath Sensor, participants were not provided access to any of the other aspects of the Pivot cessation program.

**Figure 1:**
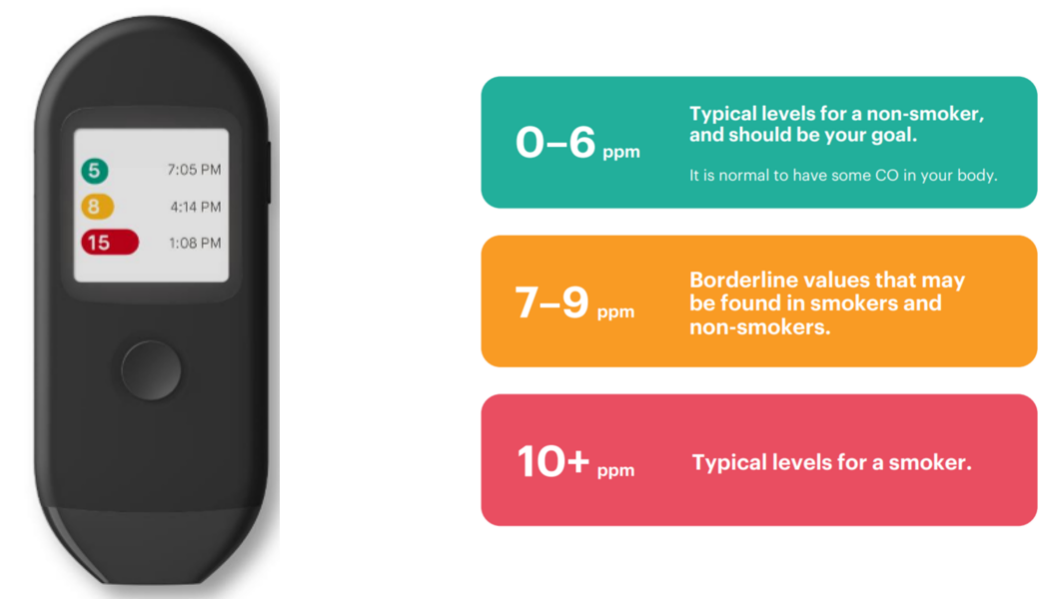
Pivot Breath Sensor and color coding of carbon monoxide values.

The Pivot Breath Sensor (Figure 1) comprises a personal interactive breath sensor that measures the level of CO in exhaled breath and displays the CO value (ppm) to the user directly on the device screen. The CO log is accessed from the sensor screen and shows the most recent exhaled breath CO value at the top of the screen. The user can view previous values by scrolling within the log. The sensor is portable, battery-powered, and rechargeable using a micro-USB cable. The user submits a breath sample by exhaling (blowing) into the breath sensor mouthpiece. CO values are color coded with the color levels (red: ≥10 ppm, orange: 7-9 ppm, green: 0-6 ppm) detailed in the labeling (Figure 1).

### Eligibility

The anticipated user population for the Pivot Breath Sensor are lay users who are smokers, aged between 18 and 80, and capable of using a smartphone and basic smartphone apps. As such, study participant inclusion criteria included all of the following: 18-80 years of age, current daily cigarette smokers (≥10 CPD), resident of the United States, able to read and comprehend English, owns and uses a smartphone compatible with the study app (iPhone 5 and above with iOS 11 and above, or an Android smartphone with Android 5.0 and above), and willing to sign the informed consent form. Exclusion criteria included pregnancy (self-reported) or participation in a previous study sponsored by Carrot Inc.

The study employed nonproportional quota sampling (Table 1) with the aim of enrolling a study population that reflects the expected initial intended user population.

**Table 1:** Nonproportional quota sampling enrollment: targeted proportions.

Category and subcategory		Targeted %
**Age (years)**		
	18-29	≤20
	30-60	≥70
	61-80	≤10
**Cigarettes per day (CPD)**		
	10-19	40-60
	≥20	40-60
**Stage of Change^a^**		
	Intend to quit within 30 days	≥20
	Intend to quit within 6 months	≥20
	Not thinking of quitting	<20
**Gender**		
	Female	40-60
**Employment**		
	Unemployed	4-8^b^
	Employed <20 hours/week	Remainder of sample
	Employed ≥20 hours/week	Remainder of sample

^a^Stage of Change question and answer choices: Are you seriously thinking of quitting smoking? (1) Yes, within the next 30 days; (2) Yes, within the next 6 months; (3) No, not thinking of quitting.

^b^The employment rate among study participants was sought to align with the employment rate among the general US population at the time of protocol submission, which was 3.7% [26].

### Recruitment

Participants were recruited in the United States from September 2019 through November 2019 using web media (Facebook). Potential participants were asked to provide contact information, and answer questions on demographics, smartphone ownership, and smoking behavior using the online screening form. Study staff reviewed each potential participant’s responses. All study participants underwent a screening phone call where study eligibility was confirmed. Potential participants were called on a first-come-first-served basis with nonproportional quota sampling enrollment guidelines applied. During this call, study personnel informed the potential participant of the study details and answered any questions. Eligible potential participants were offered the opportunity to participate in the study. Potential participants interested in proceeding were emailed the electronic informed consent form. Upon completion, participants completed a baseline questionnaire and were mailed the Pivot Breath Sensor. Participants were considered enrolled after electronically completing the informed consent form and baseline questionnaire, pairing their breath sensor to the study app on their smartphone, and completing their first breath sample.

### Study Procedure

Participants self-trained on the Pivot Breath Sensor using the device labeling, which included product packaging, a Quick Start Guide, and package insert. In addition, participants were asked to load the study app on their smartphone. This app served as a means to sync breath sample data from the breath sensor and transmit these data to the study team. Participants had access to a technical support phone line and online user manual. A member of the study staff or customer support optionally called, texted, or emailed the participant to assist in device setup if needed. The participant initiated using the breath sensor. The enrollment date was considered study day 1.

Study participants were instructed to use the breath sensor daily for the duration of the study, with a recommendation of completing 4 or more breath samples a day, spread over the course of the day. This suggested use pattern was provided in the labeling materials and during the screening phone call but breath sensor use was ultimately at the discretion of the participant. Participants received up to twice weekly SMS text message instructions to sync their breath sensor data using the study app. No recommendations were made to participants regarding smoking behavior, and the device and study were not positioned as a smoking cessation program with participants.

Participants received periodic electronic questionnaires via email (SurveyMonkey) that focused on attitudes toward quitting, smoking behavior, and use experience with focus on impact and associated learning. There were 7 questionnaires in total, emailed at baseline, and study days 7 (1 week), 14 (2 weeks), 21 (3 weeks), 28 (4 weeks), 56 (8 weeks), and 84 (12 weeks). Participants received periodic reminders from study staff to complete the questionnaires via email, SMS text messages, or phone, as needed. On study day 84, participants received the final questionnaire and were asked to send the Pivot Breath Sensor back using a provided prepaid mailer.

Participants were compensated for collecting breath samples (US $5/day for every day in which ≥4 breath samples were collected during the first 28 days of the study and thereafter US $10/week for up to 8 weeks in which ≥20 breath samples/week were collected; up to US $220 in total for 84 days of breath sampling), completing the online questionnaires (US $10-50/questionnaire; up to US $220 in total for 7 questionnaires), and for returning the Pivot Breath Sensor (US $50). Participants could earn up to US $490 in total. Compensation was in the form of Visa gift cards that were mailed to participants. Payments were bundled over 5 payments and took 2-3 weeks to arrive to the participant after being ordered.

### Data Collection

Data collection took place on electronic case report forms completed by study participants via SurveyMonkey, and through data collected in a study app which was paired to the breath sensor via Bluetooth. Breath sensor usage data and CO results populated the app. The study team periodically synchronized and uploaded logs from the sensors during the study, which was enabled by the study app.

### Outcomes

Study outcomes focused on 3 areas: attitudes toward quitting smoking, smoking behavior, and use experience. Attitudes toward quitting smoking included Stage of Change, desire to quit (yes/no), readiness to quit, (scale 1-10), confidence to quit (scale 1-10), difficulty to quit (scale 1-10), and goals (multiple choice, 5 options; Table 2).

**Table 2:** Measures assessing attitudes toward quitting smoking.

Question	Answer Options/Scale
Are you seriously thinking of quitting smoking? (Stage of Change)	“Yes, within the next 30 days” or “Yes, within the next 6 months” or “No, not thinking of quitting”
Would you like to completely stop smoking cigarettes?	“Yes” or “No”
How ready are you to quit smoking?	Scale 1-10 (1=Not at all ready, 10=Completely ready)
If you were to quit smoking right now, how successful would you be?	Scale 1 to 10 (1=Not at all successful, 10=Completely successful)
If you were to quit smoking right now, how difficult do you think it would be to stay smoke free?	Scale 1 to 10 (1=Really hard to stay quit, 10=Really easy to stay quit)
What is your goal when it comes to smoking?	“I don’t have a clear goal in mind” or “I want to quit smoking for good, even though I might slip up” or “I want to quit smoking for good” or “I want to reduce my smoking (like smoking less, or quitting for a while and deciding later if I want to quit)” or “Other goal _______________________”

Smoking behavior outcomes comprised quit attempts, change in CPD, proportion who reduced CPD by 50% or more, and smoking cessation via 7- and 30-day PPA. A quit attempt was defined as going at least one day without smoking cigarettes, even a single puff. Participants were considered to have achieved 7-day (30-day) PPA if they answered *no* to the following question: “In the last 7 (30) days have you smoked any cigarettes, even a single puff?” As the sensor is designed and was implemented here as a tool to be used independently, without requiring face-to-face contact, and data collection was achieved through remote means using the study app and electronic questionnaires, biochemical verification of smoking status was not pursued in accordance with previous recommendations [27]. If participants reported abstinence but indicated they were smoking 1 or more CPD, they were counted as actively smoking in analyses. Finally, participants were asked to expound on their use experience by providing feedback on the impact and learning associated with the Pivot Breath Sensor.

Because previous studies on CO breath sensor use in smoking cessation have reported mixed results, some with changes in attitudes and behaviors documented early that did not persist [21,22] and some with longer-term changes observed [24,25], primary and secondary endpoints were obtained at 4 weeks in this study. These outcomes were also assessed at 12 weeks for longer-term results, which are also reported herein. Overall, endpoint assessment was designed to capture shorter- and longer-term changes, if present, as informed by previous studies.

The primary endpoint assessed change in motivation to quit smoking via response to Stage of Change at 4 weeks, compared with baseline. A positive outcome was defined as a participant responding as more motivated to quit. For example, a change in response from seriously thinking of quitting smoking “...within the next 6 months” at baseline to seriously thinking of quitting smoking “...within the next 30 days” at 4 weeks was considered a positive outcome [28].

Secondary endpoints included the proportion of participants who reported 1 or more quit attempt by 4 weeks and the proportion of participants who reduced their CPD by 50% or more by 4 weeks.

### Sample Size

Consideration for the sample size included powering the study to observe a clinically meaningful change from baseline for the primary and secondary endpoints using 80% power at a statistical significance of *P*<.05. The primary endpoint was informed by preliminary data from a similar 37-participant pilot study (data not shown). Match-paired data from the 35 participants who completed the 14-day timepoint questionnaire showed that motivation to quit, via Stage of Change, had increased in 31% (11/35), remained unchanged in 66% (23/35), and decreased in 3% (1/35) of participants from baseline. To detect a statistically significant change of these proportions in this study would require enrolling 50 participants.

The estimated proportion of participants achieving 1 or more quit attempt was 25% based on interim results from the aforementioned pilot study. Based on the median prevalence of 65.4% for past-year quit attempts in the general population [29], the average monthly quit attempt rate is approximately 5%. In the context of a 4-week outcome, to show that 25% of participants making a quit attempt by 4 weeks is statistically different from 5% would require enrolling 16 participants.

Finally, previous work indicates approximately 1% of the general population [30-34] and 2%-5% of individuals in cigarette reduction studies [35,36] will reduce their CPD by 50% or more on a monthly basis. Assuming that 10% of participants in this study will reduce their CPD by 50% or more by 4 weeks would require enrolling 185 participants to show a difference from 5%. Taking these analyses into consideration along with expected attrition, this study targeted enrollment of 220 participants.

### Statistical Analysis

Changes in measurements from baseline were assessed at different timepoints in the study. Participants served as their own controls and tests for any change were performed. Analyses were conducted to calculate mean (SD) for normally distributed variables for actual data, or mean (SE) for modeled data. Median (IQR) values were used in instances of non-normally distributed variables. As applicable, paired one-sample *t*-test was used for numeric data. For one-sample change in binary outcomes, compared binomial proportion test was applied. Fisher exact or chi-square tests were used for categorical data. McNemar test was applied for 2-category match-paired data. Stuart–Maxwell test was used for 3-category match-paired data. To evaluate changes in attitudes or changes in CPD over time, repeated measures linear mixed model analyses were performed using a compound symmetric correlation matrix to model the repeated measures within subjects. To make specific comparisons across time, *F* statistics were computed using the results from the model. Analyses were conducted using SAS version 9.4 (SAS Institute). Statistical significance was set at *P*<.05.

Analyses for the outcomes were evaluated for only those who completed the questionnaire (ie, completer) and assessed to take into account missing data. To be included in any completer analysis the participant must have provided a baseline response and the outcome response. For outcomes that were missing data, these were assessed pending the data type. For categorical data, the last response postbaseline and pre-4 weeks was carried forward for the 4-week assessment, and the last response postbaseline and pre-12 weeks was carried forward for the 12-week assessment. If there were no data to carry forward, the data were considered unchanged from baseline. Numeric data used linear mixed modeling.

## Results

### Enrollment

**Figure 2:**
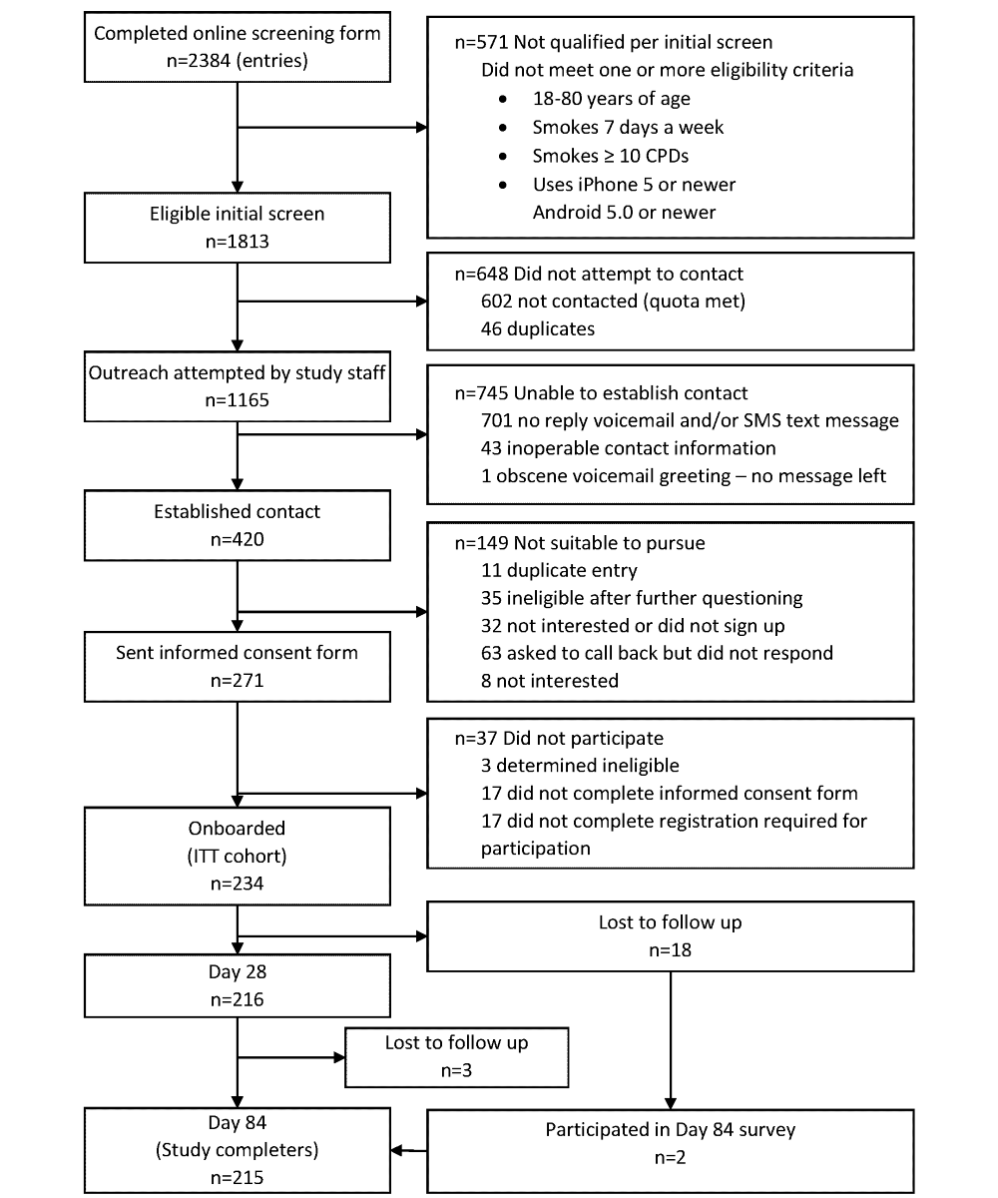
Study participant flow: Consolidated Standards of Reporting Trials (CONSORT) diagram.

Potential participants responded to an online Facebook ad that included a link to the online screening form. The online screening form was completed by 2384 individuals, of whom 1813 were eligible for the study. Eligible potential participants were called on a first-come-first-served basis with nonproportional quota sampling enrollment guidelines applied; outreach was made to 1165 potential participants. The majority of phone calls went unanswered and unreturned; contact was established with 420 individuals. The electronic informed consent form was sent to 271 individuals, 234 of whom enrolled in the study. Study participant flow is depicted via a Consolidated Standards of Reporting Trials (CONSORT) diagram in Figure 2.

The study overenrolled by 6.4% (14/220 participants) with a final enrollment of 234 participants. Because of the multistep process of enrollment and expected attrition over the course of the study, we put forth a good faith effort to ensure we obtained data from 185 participants at 4 weeks. The results of these efforts yielded this slight overenrollment. Considering the minimal risk profile of the device and the voluntary, ambulatory nature of the study in which participants gave breath samples and completed online questionnaires at their discretion, this overenrollment was not felt to be significant.

The study sample consisted of 52.6% women (123/234), had a mean age of 39.9 (SD 11.3) years, smoked a mean of 20.3 (SD 8.0) CPD at baseline, and had been smoking for a mean of 21.7 (SD 11.5) years.

At baseline, 15.4% (36/234) indicated they were seriously thinking of quitting smoking in the next 30 days, 76.9% (180/234) indicated they were thinking of quitting in the next 6 months, and 7.7% (18/234) indicated they were not seriously thinking of quitting smoking. On average, participants had made 2.1 (SD 6.3) quit attempts over the past 12 months.

All nonproportional quota sampling targets were achieved with the exception of 20% or more (≥47/234) indicating they intended to quit smoking in the next 30 days. This differential is due to change in participant response to this question between when it was asked on the online screening form and when it was asked on the baseline questionnaire. Specifically, 29.1% (68/234) of study participants indicated they were seriously thinking of quitting smoking in the next 30 days on the online screening form; however, this decreased to 15.4% (36/234) of participants on the baseline questionnaire. This difference was not felt likely to significantly affect outcomes. All individuals who changed their response went from, “Seriously thinking of quitting in the next 30 days” at online screening to “Seriously thinking of quitting in the next 6 months” at baseline, thereby maintaining some interest in quitting smoking, albeit on a longer timeline. If anything, the effect of enrolling a cohort slightly less motivated to quit than originally anticipated may have made it more challenging to achieve some outcomes, such as quit attempt rates and change in CPD. Study demographic details, including targeted and actual nonproportional quota proportions, are provided in Table 3.

**Table 3:** Baseline demographics including targeted and actual nonproportional quota sampling (N=234, when applicable).

Characteristic		Values^a^	Target %
**Gender**			
	Male	111 (47.4)	40-60
	Female	123 (52.6)	40-60
**Age (years), mean (SD)**		39.9 (11.3)	
	18-29 years	37 (15.8)	≤20
	30-60 years	185 (79.1)	≥70
	61-80 years	12 (5.1)	≤10
**Ethnicity**			
	White	209 (89.3)	
	Hispanic, Latinx, or Spanish Origin	5 (2.1)	
	Black or African American	9 (3.8)	
	Asian	3 (1.3)	
	American Indian or Alaska Native	2 (0.9)	
	Middle Eastern or North African	1 (0.4)	
	Hawaiian or Other Pacific Islander	0 (0)	
	Some other race, ethnicity, or origin	5 (2.1)	
	Prefer not to answer	0 (0)	
**US region**			
	Northeast	34 (14.5)	
	South	112 (47.9)	
	Midwest	48 (20.5)	
	West	40 (17.1)	
**Education**			
	Less than 8th grade	0 (0)	
	Some high school	7 (3.0)	
	High school/General educational development	50 (21.4)	
	Some college	107 (45.7)	
	Associate’s (2-year) degree	37 (15.8)	
	Bachelor’s (4-year) degree	25 (10.7)	
	Master’s degree	7 (3.0)	
	Professional or doctorate degree	1 (0.4)	
**Employment**			
	Unemployed	11 (4.7)	4-8
	Employed <20 hours/week	29 (12.4)	Remainder of sample
	Employed ≥20 hours/week	194 (82.9)	Remainder of sample
**Household income**			
	<US $25,000	53 (22.6)	
	US $25,000 to US $34,999	53 (22.6)	
	US $35,000 to US $49,999	47 (20.1)	
	US $50,000 to US $74,999	42 (17.9)	
	US $75,000 to US $99,999	18 (7.7)	
	US $100,000 to US $149,999	14 (6.0)	
	≥US $150,000	4 (1.7)	
	Prefer not to answer	3 (1.3)	
**Smartphone type**			
	iPhone	59 (25.2)	
	Android	175 (74.8)	
Years smoking, mean (SD)		21.7 (11.5)	
**Cigarettes per day (CPD), mean (SD); range (min-max)**		20.3 (8.0); 7-50	
	<20 CPD	107 (45.7)	40-60
	≥20 CPD	127 (54.3)	40-60
**How soon after waking up do you typically smoke your first cigarette?**			
	Within 5 minutes	114 (48.7)	
	5 to 30 minutes	99 (42.3)	
	31 to 60 minutes	13 (5.6)	
	60+ minutes	8 (3.4)	
**Would you like to completely stop smoking cigarettes?**			
	Yes	217 (92.7)	
	No	17 (7.3)	
**Motivation to Quit (Stage of Change)—Are you seriously thinking of quitting smoking?**			
	Yes, within the next 30 days	36 (15.4)	≥20
	Yes, within the next 6 months	180 (76.9)	≥20
	No, not thinking of quitting	18 (7.7)	<20
**What is your goal when it comes to smoking?**			
	I don’t have a clear goal in mind	40 (17.1)	
	I want to quit smoking for good, even though I might slip up	75 (32.1)	
	I want to quit smoking for good	84 (35.9)	
	I want to reduce my smoking (like smoking less, or quitting for a while and deciding later if I want to quit)	34 (14.5)	
	Other goal	1 (0.4)	
Quit attempts over past 12 months^b^, mean (SD)		2.1 (6.3)	
**Use of other tobacco products**			
	Cigars, cigarillos or little filtered cigars	31 (13.2)	
	A regular pipe	0 (0)	
	Hookah or water pipe	5 (2.1)	
	E-cigarettes or vape	67 (28.6)	
	Smokeless tobacco, chew, or snuff	8 (3.4)	
Readiness to quit^c^, mean (SD)		5.6 (2.7)	
Success to quit^d^, mean (SD)		3.3 (2.3)	
Difficulty to quit^e^, mean (SD)		2.8 (2.4)	
First carbon monoxide measurement (ppm)^f^, mean (SD)		26.7 (18.4)	

^a^All data are presented as n (%) unless otherwise indicated.

^b^Quit attempt=“How many times have you tried to quit smoking where you’ve gone at least one day without smoking a cigarette, even a single puff?”

^c^How ready are you to quit smoking (1=Not at all ready, 10=Completely ready).

^d^If you were to quit smoking right now, how successful would you be? (1=Not at all successful, 10=Completely successful).

^e^If you were to quit smoking right now, how difficult do you think it would be to stay smoke free? (1=Really hard to stay quit, 10=really easy to stay quit).

^f^ppm: parts per million.

### Attitudes Toward Quitting Smoking

For the primary endpoint, motivation to quit smoking at 4 weeks was significantly increased compared with baseline as measured with Stage of Change (Table 4). Motivation to quit smoking improved with 38.9% (84/216) of respondents indicating they were seriously thinking of quitting in the next 30 days compared with 14.4% (31/216) at baseline (*P*<.001). At 4 weeks, motivation to quit smoking increased in 29.6% (64/216), was unchanged in 66.7% (144/216), and decreased in 3.7% (8/216; *P*<.001).

**Table 4:** Change in motivation to quit smoking (N=216) at baseline (rows) versus 4 weeks (columns).

		Motivation to Quit: 4 weeks, n (%)				
		Yes, within the next 30 days	Yes, within the next 6 months	No, not thinking of quitting	Total^b^	
**Motivation to Quit** ^a^ **: Baseline**						
	Yes, within the next 30 days, n (%)	25 (11.6)	6 (2.8)	0 (0)	31 (14.4)	
	Yes, within the next 6 months, n (%)	58 (26.9)	108 (50.0)	2 (0.9)	168 (77.8)	
	No, not thinking of quitting, n (%)	1 (0.5)	5 (2.3)	11 (5.1)	17 (7.9)	
	Total, n (%)^b^	84 (38.9)	119 (55.1)	13 (6.0)	216 (100.0)^c^	

^a^Motivation to quit smoking assessed via Stage of Change question: “Are you seriously thinking of quitting smoking?” (1) “Yes, within the next 30 days”; (2) “Yes, within the next 6 months”; (3) “No, not thinking of quitting.”

^b^*P*<.001.

^c^A total of 234 participants enrolled in the study; however, only 216 completed the 4-week questionnaire, who are represented here.

Assuming a worst case scenario in which the 18 participants who did not complete the 4-week questionnaire had the lowest possible motivation to quit smoking at 4 weeks (No, not thinking of quitting), 35.9% (84/234) of respondents would have fallen in the category of “seriously thinking of quitting in the next 30 days,” compared with 15.4% (36/234) at baseline (*P*<.001). Motivation to quit smoking would have increased in 27.4% (64/234), remain unchanged in 62.0% (145/234), and decreased in 10.7% (25/234; *P*<.001).

There were further increases in motivation at 12 weeks. Among the 215 study participants who completed the 12-week questionnaire, motivation to quit smoking improved, with 47.9% (103/215) of respondents indicating they were seriously thinking of quitting in the next 30 days compared with 14.9% (32/215) at baseline (*P*<.001). Motivation to quit smoking increased in 39.1% (84/215), was unchanged in 54.9% (118/215), and decreased in 6.0% (13/215; *P*<.001).

Similar to the previous worst case analysis, if all 19 participants who did not complete the 12-week questionnaire had the lowest possible motivation to quit smoking (No, not thinking of quitting), 44.0% (103/234) of respondents would have fallen in the category of “seriously thinking of quitting in the next 30 days,” compared with 15.4% (36/234) at baseline (*P*<.001). Motivation to quit smoking would have increased in 35.9% (84/234), remain unchanged in 50.9% (119/234), and decreased in 13.2% (31/234; *P*<.001).

Participants were asked if they would like to stop smoking. Matched responses at baseline and at 12 weeks are shown in Table 5. The majority (>90%, >197/215) of participants indicated they would like to stop smoking at baseline and at 12 weeks, with 12/216 people (5.6%) changing their response: 5 from *yes* to *no* and 7 from *no* to *yes* (*P*=.77).

**Table 5:** Would you like to completely stop smoking cigarettes (N=215)? Select one.

			12 weeks, n (%)				
			Yes		No		Total^a^
**Baseline**							
	Yes, n (%)	193 (89.8)		5 (2.3)		198 (92.1)	
	No, n (%)	7 (3.3)		10 (4.7)		17 (7.9)	
	Total^a^, n (%)	200 (93.0)		15 (7.0)		215^b^ (100.0)	

^a^*P*=.77.

^b^234 participants enrolled in the study; however, only 215 completed the 12-week questionnaire, who are represented here.

**Figure 3:**
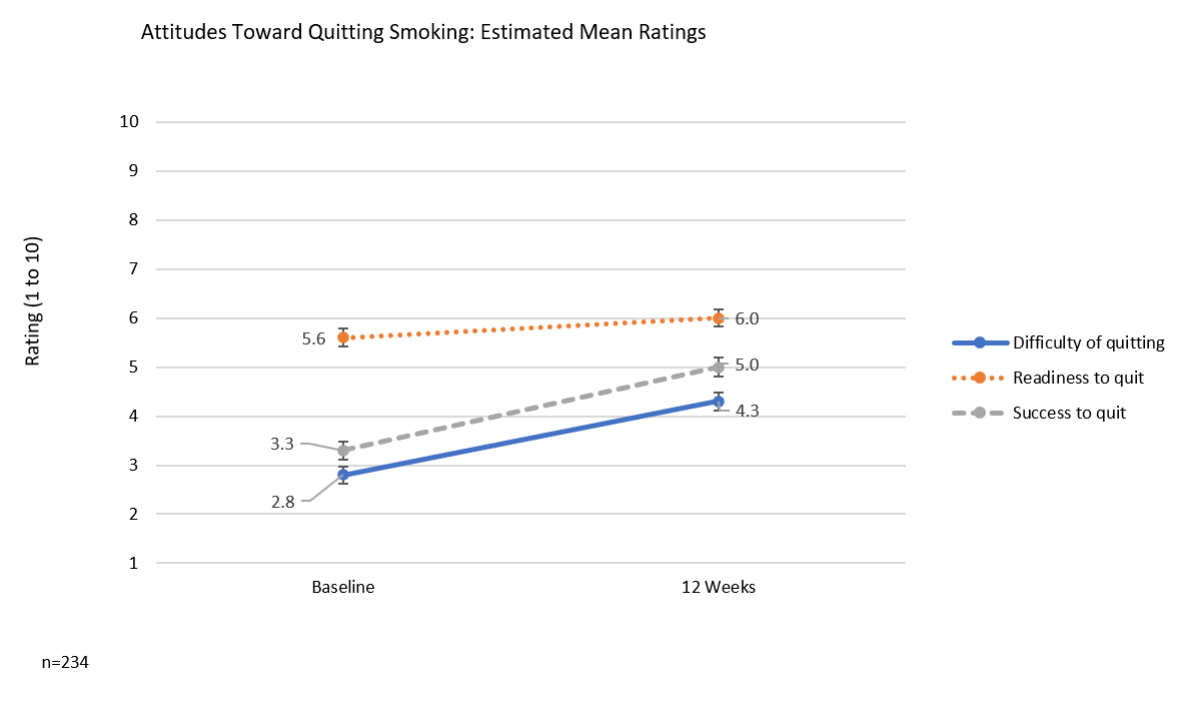
Attitudes towards quitting smoking, ratings (scale 1-10) at baseline vs. 12 weeks. Estimate of means and standard errors based on linear mixed model. Readiness to quit smoking (RTQ), Difficulty to quit smoking (DTQ), Success to quit smoking (STQ).

Assessment of readiness to quit, success to quit, and perceived difficulty of quitting at baseline versus 12 weeks is depicted in Figure 3. Using linear mixed models to include intervening timepoints, the readiness to quit at 12 weeks was estimated not to change (5.6 vs 6.0; *P*=.07). By contrast, the estimated ratings for success to quit and difficulty of quitting were greater than the estimated baseline values at 3.3 versus 5.0 (*P*<.001) and 2.8 versus 4.3 (*P*<.001), respectively.

**Figure 4:**
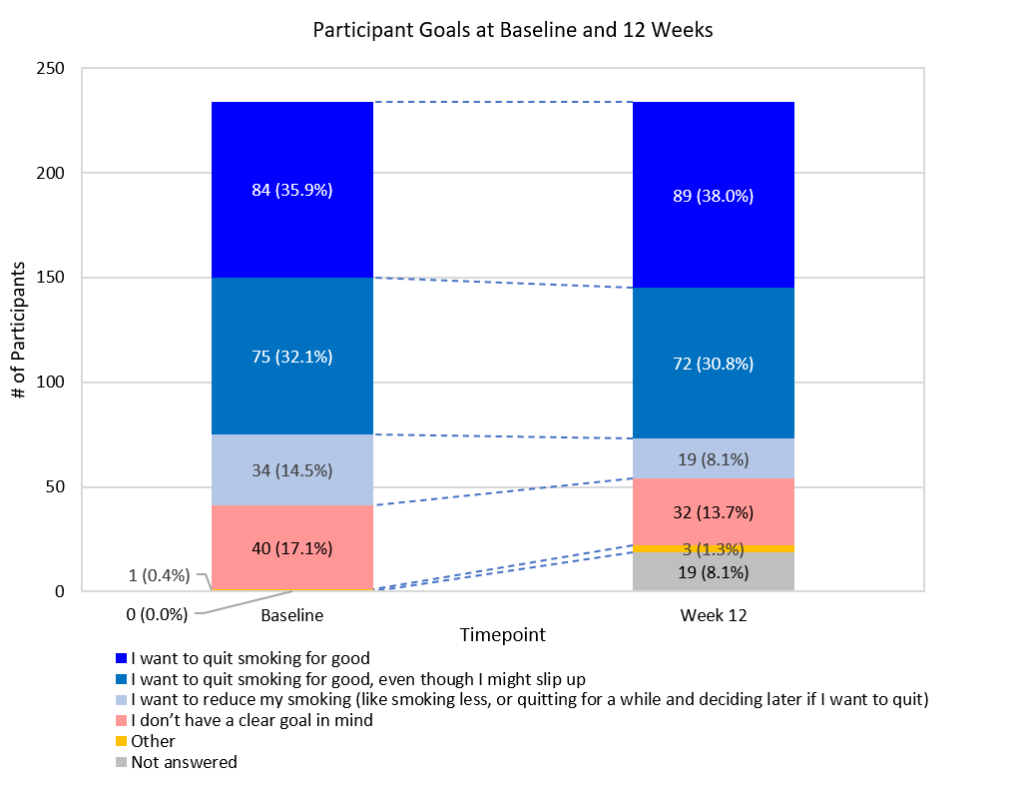
Participant goals at baseline and 12 weeks.

Participants were asked their goal as it relates to smoking. Overall, the proportion of the cohort in each goal category was stable at both timepoints, with a slight increase in the proportion indicating they wanted to quit for good and a slight decrease in the proportion indicating they want to reduce their smoking (Figure 4) at 12 weeks. Matched pair data are detailed in Table 6. Excluding the 4 participants who answered *Other*, 28.0% (59/211) strengthened their goal toward quitting, 55.0% (116/211) maintained their goal, and 17.1% (36/211) weakened their goal.

**Table 6:** Participant goals at baseline (rows) and 12 weeks (columns), match-paired analysis (N=215).

		Goals: 12 weeks						
		Quit smoking for good	Quit smoking for good, even though I might slip up	Reduce my smoking	No clear goal in mind	Other	Total	
**Goals: Baseline**								
	Quit smoking for good, n (%)	55 (25.6)	15 (7.0)	2 (0.9)	6 (2.8)	0 (0.0)	78 (36.3)	
	Quit smoking for good, even though I might slip up, n (%)	20 (9.3)	38 (17.7)	1 (0.5)	7 (3.3)	2 (0.9)	68 (31.6)	
	Reduce my smoking, n (%)	5 (2.3)	13 (6.0)	9 (4.2)	5 (2.3)	0 (0.0)	32 (14.9)	
	No clear goal in mind, n (%)	9 (4.2)	6 (2.8)	6 (2.8)	14 (6.5)	1 (0.5)	36 (16.7)	
	Other, n (%)	0 (0.0)	0 (0.0)	1 (0.5)	0 (0.0)	0 (0.0)	1^a^ (0.5)	
	Total, n (%)	89 (41.4)	72 (33.5)	19 (8.8)	32 (14.9)	3^b^ (1.4)	215 (100.0)	

^a^Of the 234 participants who completed the baseline questionnaire, 1 person selected *Other* for their goal and wrote, “Smoke less at this time.”

^b^Of the 215 participants who completed the 12-week questionnaire, 3 people selected *Other* for their goal and wrote: (1) “Already quit”; (2) “Some of these questions become irrelevant once you quit smoking”; (3)“Already have stopped.”

### Smoking Behavior

For secondary endpoints, 28.2% (66/234, ITT; 95% CI 22.5%-34.4%) reported making 1 or more quit attempt, and 23.1% (54/234, ITT; 95% CI 17.8%-29.0%) reduced their CPD by 50% or more by 4 weeks.

Quit attempt and CPD reduction rates increased over time. At 12 weeks, 48.3% (113/234, ITT; 95% CI 41.7%-54.9%) reported making 1 or more quit attempt, with mean 2.4 (SD 9.1; CI 1.2-3.6) quit attempts per participant. Overall, CPD reduction of 50% or more occurred in 38.5% (90/234, ITT; 95% CI 32.2%-45.0%) of participants. Among the study completers who did not achieve at least seven-day PPA, 33.2% (62/187, completer; 95% CI 26.5%-40.4%) reduced their CPD by 50% or more.

**Figure 5:**
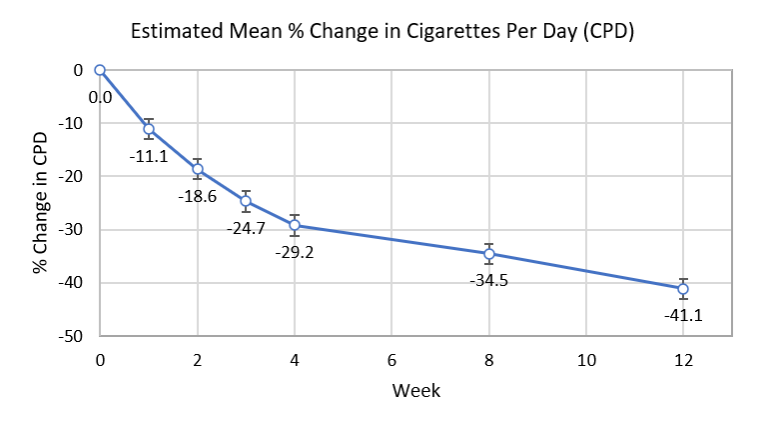
Percent change in cigarettes per day (CPD) over time. Estimate of means and standard errors based on linear mixed model.

By 12 weeks, 82.8% (178/215) of participants had reduced CPD, 11.6% (25/215) had no change, and 5.6% (12/215) had increased CPD. Linear mixed model analysis was performed, with projected mean CPD values at various timepoints compared with baseline and each other. Mean CPD decreased steadily over the course of the study, with the most pronounced drop over the first 4 weeks (Figure 5). At 12 weeks, mean CPD was reduced by 41.1% compared with baseline. Decreases in CPD were statistically significant at each timepoint (all *P*<.001, but *P*=.001 for 3 weeks vs 4 weeks). Among the study completers who did not achieve at least seven-day PPA (n=187), CPD were reduced by 32.3% at 12 weeks.

At 12 weeks, 7-day PPA was 12.0% (28/234, ITT) and 30-day PPA was 6.0% (14/234, ITT). Analysis of those who completed the study (n=215) yields a 7-day PPA of 13.0% (28/215, completer) and a 30-day PPA of 6.5% (14/215, completer).

### Use Experience

Over the course of the study, use experience was assessed via participant feedback on the impact of the breath sensor on attitudes toward quitting smoking and smoking behavior, and on learning associated with breath sensor use.

**Figure 6:**
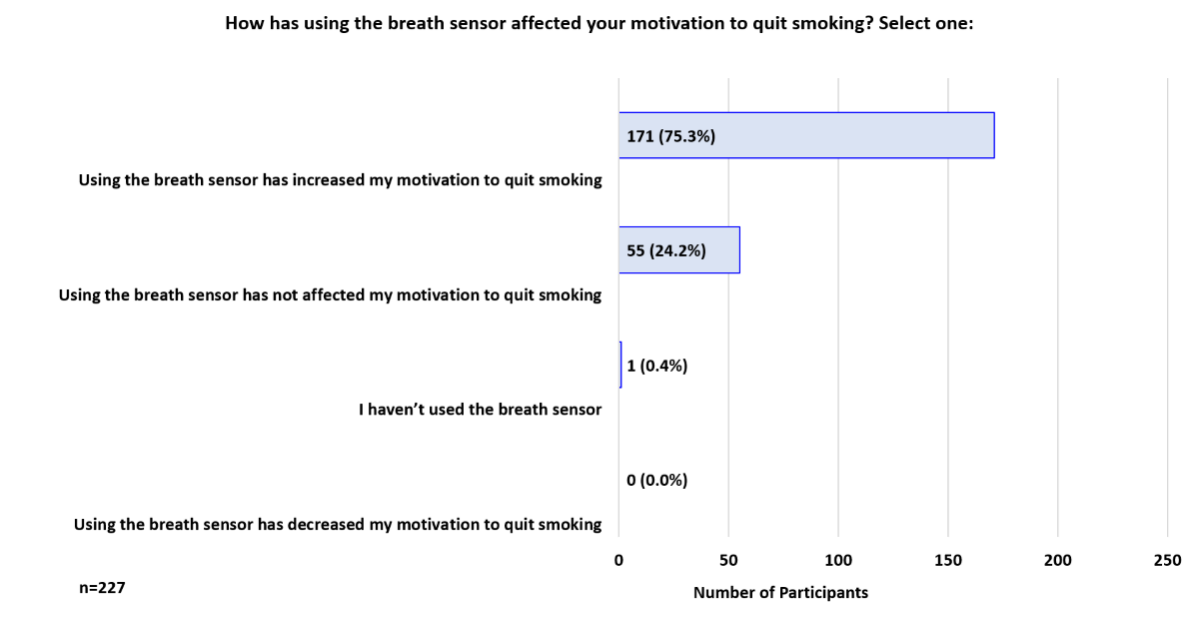
Participant Feedback: Effect of breath sensor on motivation to quit smoking (week 1).

**Figure 7:**
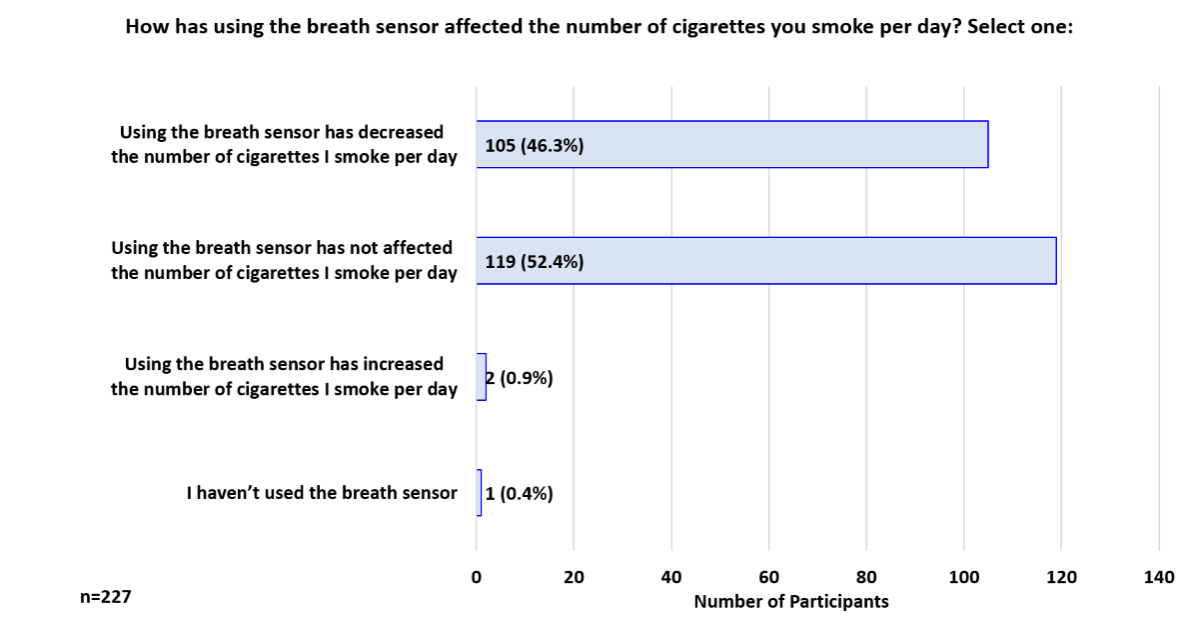
Participant Feedback: Effect of breath sensor on number of cigarettes smoked per day (week 1).

**Figure 8:**
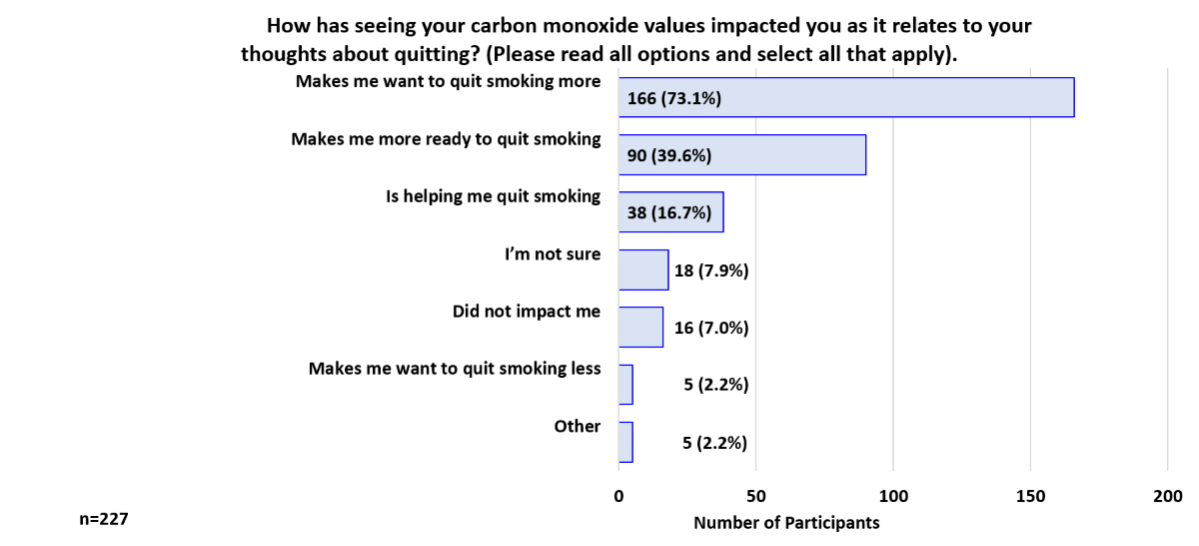
Participant Feedback: Impact of carbon monoxide values on thoughts about quitting (week 1).

At 1 week, 75.3% (171/227) reported that using the breath sensor increased their motivation to quit smoking (Figure 6). When asked how using the breath sensor had affected the number of cigarettes smoked per day, 52.4% (119/227) responded it had not affected their CPD, while 46.3% (105/227) indicated it had decreased their CPD (Figure 7). Participants were asked how seeing their CO values had impacted their thoughts about quitting smoking; the top 3 responses were: “Makes me want to quit smoking more” 73.1% (166/227), “Makes me more ready to quit smoking” 39.6% (90/227), and “Is helping me quit smoking” 16.7% (38/227; Figure 8).

**Figure 9:**
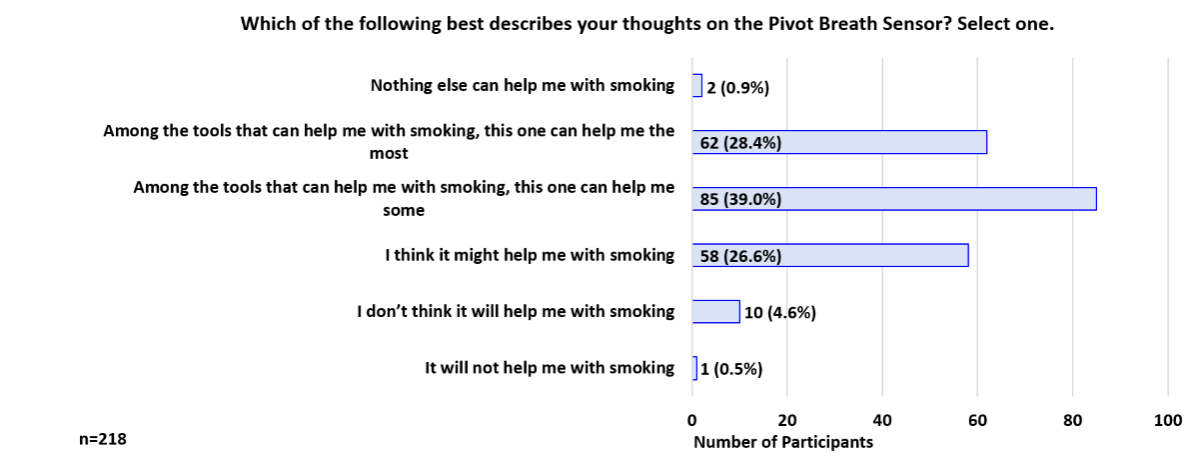
Participant Feedback: Thoughts on the Pivot Breath Sensor (week 2).

**Figure 10:**
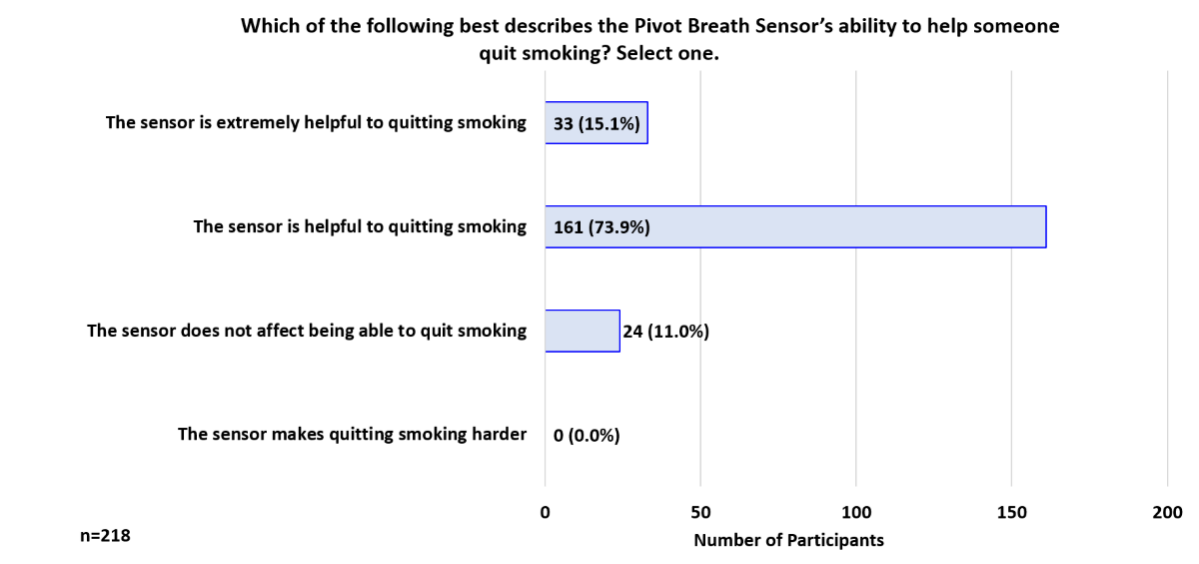
Participant Feedback: Pivot Breath Sensor’s ability to help someone quit smoking (week 2).

At 2 weeks, when asked which statement best describes their thoughts on the breath sensor, 39.0% (85/218) indicated, “Among the tools that can help with smoking, this one can help me some” and 28.4% (62/218) indicated, “Among the tools that can help me with smoking, this one can help me the most” (Figure 9). In addition, 89.0% (194/218) indicated the sensor is *extremely helpful* or *helpful* in helping someone quit smoking (Figure 10).

**Figure 11:**
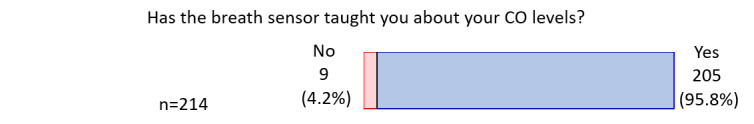
Participant Feedback: Has the breath sensor taught you about your carbon monoxide (CO) levels? (week 3).

**Figure 12:**
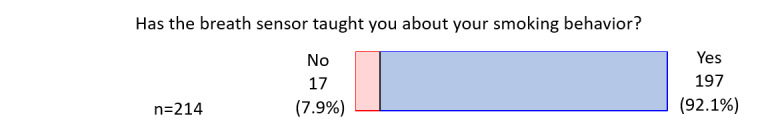
Participant Feedback: Has the breath sensor taught you about your smoking behavior? (week 3).

At 3 weeks, the majority (>90%, >196/214) of participants indicated the breath sensor had taught them about their CO levels and smoking behavior (Figures 11 and 12).

**Figure 13:**
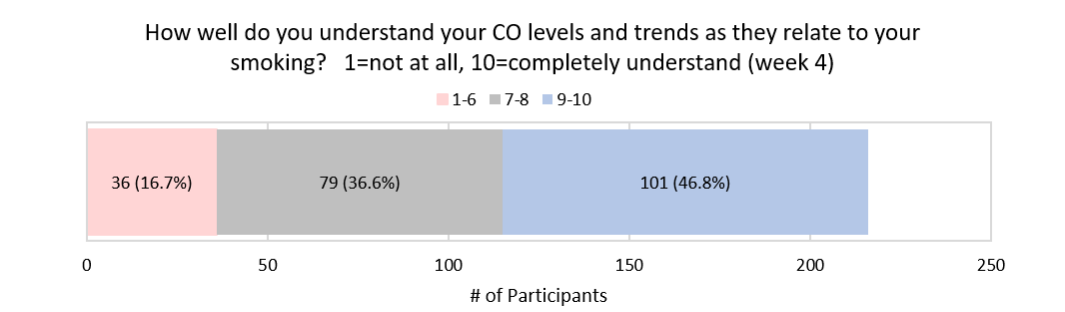
Participant Feedback: Understanding of CO levels and trends as they relate to smoking behavior (week 4).

At 4 weeks, on a scale of 1-10, the mean score for how well participants understood their CO levels and trends as they relate to their smoking behavior was 8.0 (SD 2.1; Figure 13).

**Figure 14:**
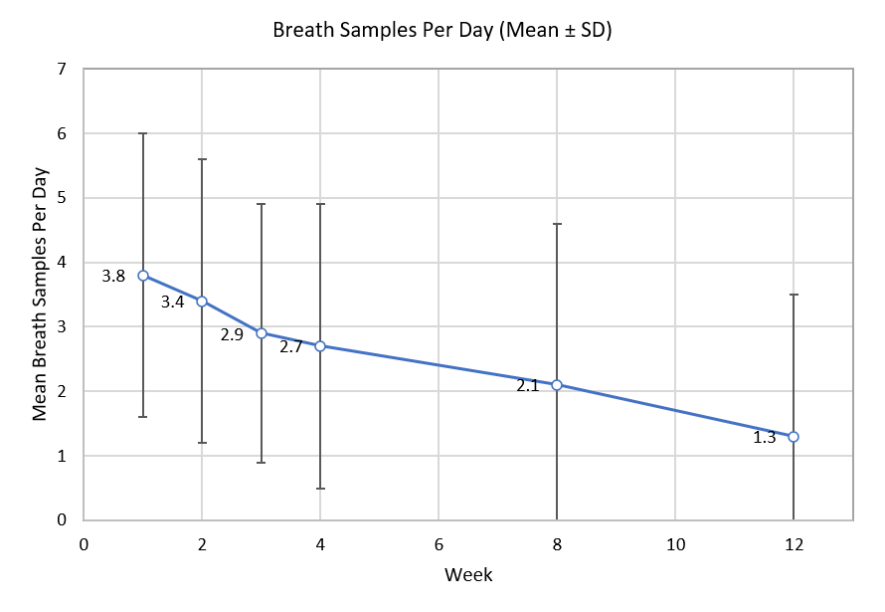
Breath sensor use.

Assessment of participant breath sensor use revealed regular daily use that decreased over time, with a mean of 3.8 (SD 2.2) samples per day at 1 week compared with 1.3 (SD 2.2) at 12 weeks (Figure 14). Overall, a total of 48,747 breath samples were taken over the course of the study with each participant performing a mean of 208.3 (SD 141.8) total breath samples.

**Figure 15:**
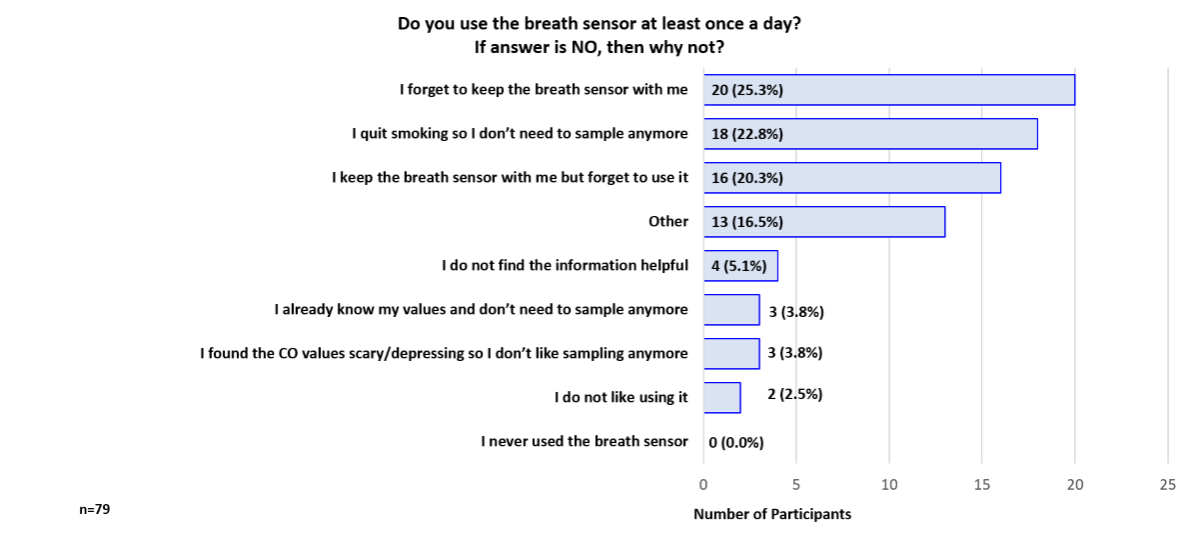
Participant Feedback: Reasons for not using the breath sensor (week 12).

At 12 weeks, among the 79 participants who indicated they were not using the breath sensor at least once per day, the top 3 reasons were “I forget to keep the breath sensor with me” (25% 20/79), “I quit smoking so I don’t need to sample anymore” (23%, 18/79), and “I keep the breath sensor with me but forget to use it” (20%, 16/79; Figure 15).

### Adverse Events

There were no adverse events.

## Discussion

### Principal Findings

This study evaluated attitudes toward quitting smoking, smoking behavior, and use experience in 234 adult smokers using the Pivot Breath Sensor over a 12-week period. Participants had a significant increase in motivation to quit smoking as assessed through Stage of Change (*P*<.001). Specifically, motivation to quit smoking increased in 29.6% (64/216) by week 4, and in 39.1% (84/215) by week 12. Positive changes in smoking behavior occurred as well; 28.2% (66/234) made 1 or more quit attempt and 23.1% (54/234) reduced their CPD by 50% or more at 4 weeks, which increased to 48.3% (113/234) and 38.5% (90/234), respectively, at 12 weeks. Among those who completed the 12-week questionnaire, 82.8% (178/215) had reduced CPD with an average CPD reduction of 41.1%. Moreover, 12.0% (28/234) achieved 7-day PPA and 6.0% (14/234) achieved 30-day PPA. Additional measures of attitudes toward quitting, specifically success to quit and perceived difficulty of quitting, had significant improvement (*P*<.001). In assessing use experience, 75.3% (171/227) reported that using the breath sensor increased their motivation to quit smoking. Most participants (>90%, >196/214) indicated the breath sensor taught them about their CO levels and smoking behavior. A majority of participants (73.1%, 166/227) indicated that seeing their CO values made them want to quit smoking more.

### Evidence-Based Context of Outcomes

Assessing the results with consideration of available published data facilitates contextual interpretation of the study outcomes. Regarding the significance of increased motivation to quit smoking, Prochaska et al [28,37] reported that advancement of one stage in the Stage of Change assessment during the first month of treatment almost doubles the chances that a smoker will take effective action in the next 6 months. In addition, baseline Stage of Change predicts long-term quit rates [38,39]. For example, in an intensive action- and maintenance-oriented smoking cessation program for cardiac patients, validated abstinence from smoking at 6 months was achieved in 11% of those not thinking of quitting smoking at baseline, 27% of those thinking of quitting in the next 6 months at baseline, and 56% of those thinking of quitting in the next 30 days or actively making a quit attempt at baseline [40]. These data underpin the value of having high or recently increased motivation at the outset of a smoking cessation program. In this study, 38.9% (84/216) of study participants were seriously considering quitting in the next 30 days at 4 weeks (up from 14.4%, or 31/216, at study entry), and 29.6% (64/216) had increased motivation to quit over the first 4 weeks of the study; these individuals now have an increased likelihood of quitting smoking.

Concerning quit attempts, based on the median prevalence of 65.4% for past-year quit attempts among adult US smokers [29], the average monthly quit attempt rate is approximately 5%. In this study, 28.2% (66/234) of participants made a quit attempt over a 1-month period, a more than fivefold increase of the general population average. This quit attempt rate is further notable when considering that at baseline, 84.6% (198/234) of the study participants were not particularly motivated to quit, indicating they were seriously thinking about quitting in the next 6 months or not thinking about quitting smoking. Quit attempts are meaningful because increases in smoking cessation are driven in large part by increases in quit attempts [41]. Accordingly, the CDC and Healthy People initiative have identified increasing quit attempts as an important goal for tobacco control efforts [5,42].

Finally, approximately 1% of the general population of smokers [30-34] and 2%-5% of individuals in cigarette reduction studies [35,36] will reduce their CPD by 50% or more on a monthly basis. In this study, 23.1% (54/234) of participants reduced their CPD by 50% or more over a 1-month period, increasing to 38.5% (90/234) at 12 weeks. Reducing CPD by 50% or more is clinically meaningful as the rates of quit attempts or cessation itself significantly increase among those who achieve this degree of CPD reduction [43].

### Comparison With Prior Work

Comparison of outcomes with previous studies is limited by differences in study design, particularly in the method and frequency of CO breath sampling, a constraint that compelled the undertaking of this study in the first place. In most previous assessments, CO breath sampling was administered by study staff or health professionals at study visits, and participants performed no more than a few breath sampling. In this study, participants used a personal interactive CO breath sensor to sample their breath multiple times per day over a 12-week period, with sampling done at the participant’s discretion. Indeed, participants sampled extensively, with each performing an average of 208.3 breath samples.

The comparator study most similar in design to this study is Beard et al’s investigation [16], in which participants used a personal CO breath sensor on an outpatient basis over a 6-week period [16]. Acknowledging that the study by Beard et al [16] was small (N=10), the results of the 2 studies are in range of each other: CPD was reduced by 32.6% at 6 weeks (Beard et al [16]) and 34.5% at 8 weeks (this study) and quit attempts were made by 50.0% (5/10) of participants at 6 weeks (Beard et al [16]) and 38.5% (90/234) at 8 weeks (this study).

The approach to CO breath sampling in Beard et al’s study [16] and this investigation enabled participants to directly link their smoking behavior to their CO values and track their progress over time. The benefit of tracking one’s behavior and progress via self-guided biofeedback, evident here in smoking behavior, is also well documented in other disease states [8-10,44-46] lending further support to this approach.

### Limitations

There are a few important limitations of this study. First, while this study reports results from a long-term use period (12 weeks) of a personal CO breath sensor, it does not include outcomes following the period where the breath sensor was used. This limits the understanding of outcome durability and highlights the need for longer-term data.

Second, participants were compensated for breath sampling. This was deliberate in this initial attempt at understanding the impact of personal mobile CO breath sampling on adult smokers. The investigators opted for an optimized use scenario, to understand outcomes in the setting of reliable and steady breath sensor use. This may limit the generalizability of the results, particularly those addressing breath sampling behavior. Future research should address how individuals behave when not incentivized to breath sample, and whether this real-world behavior yields results different from those reported herein. We did take steps to minimize the impact of compensation. First, we instituted a temporal delay between behavior and the associated payment (approximately 3 weeks). Second, payments were structured such that no individual payment was larger than US $140. Moreover, while compensation was linked to the completion of breath sampling, it was not linked to outcomes such as attitudes, smoking behavior, or the content of participant feedback. The decrease in breath sampling from an average of 3.8 samples per participant per day at 1 week to 1.3 at 12 weeks suggests the study compensation did not unduly influence breath sampling behavior.

There are additional study design considerations to address as well. First, we did not require proof that breath samples were provided only by study participants. We believe this possibility is unlikely, as breath sampling over the course of the study largely followed the expected pattern of single-person use, with decreasing number of samples over time. When designing the study, we considered the drawbacks of implementing monitoring, including further differentiating sensor use in the study from real-world sensor use experience, decreasing autonomy and convenience for participants, and instilling a sense of policing that might have affected participant perception and experience of the sensor. Nonetheless, because we cannot exclude the possibility that someone other than the participant provided breath samples, we acknowledge this as a limitation.

In addition, it is important to consider that the study design, as a prospective cohort study, limits understanding of the influence of baseline motivation to quit on participant outcomes. We do believe it is beneficial that the majority of study participants (76.9%, 180/234) were thinking of quitting in the next 6 months for 2 reasons: (1) This population had room for both observable improvement (thinking of quitting in the next 30 days) and worsening (not thinking of quitting) of motivation. (2) At this baseline level of motivation (thinking of quitting in the next 6 months), previous work indicates most were unlikely to change their smoking during the duration of the study [47]. Overall, this single-arm study was conducted as an initial assessment of personal mobile breath CO sampling in adult smokers. Now that these initial results have been established, future study via a randomized control trial is an appropriate next step. The aforementioned issues, particularly those of duration of follow-up after the period of breath sensor use and better understanding the role of baseline motivation to quit, should be addressed in any future research.

### Conclusion

In this study, smokers who used the Pivot Breath Sensor over a 12-week period had increased motivation to quit, reduced CPD, and had favorable quit attempt rates. These are meaningful milestones in the process of smoking cessation, conferring increased likelihood of success. Accordingly, the results suggest a role for personal biofeedback via mobile CO breath sampling in smoking cessation, particularly as a means to facilitate motivational advancement and favorable change in smoking behavior.

## Abbreviations

CO: carbon monoxide
CPD: cigarettes per day
ITT: intention to treat
PPA: point prevalence abstinence
ppm: parts per million
